# Moderated Role of Social Support in the Relationship between Job Strain, Burnout, and Organizational Commitment among Operating Room Nurses: A Cross-Sectional Study

**DOI:** 10.3390/ijerph191710813

**Published:** 2022-08-30

**Authors:** Na Li, Lichuan Zhang, Xuejing Li, Qian Lu

**Affiliations:** 1 School of Nursing, Shandong University of Traditional Chinese Medicine, Jinan 250355, China; 2 School of Nursing, Peking University, Beijing 100191, China; 3 Division of Operating Center, Peking University People’s Hospital, Beijing 100044, China

**Keywords:** burnout, job strain, moderated role, nursing, operating room, organizational commitment, social support

## Abstract

Unique environment, coupled with overload, low job control, and high risk might put operating room (OR) nurses in a state of high job strain, which might have negative influences on burnout and organizational commitment. Based on the Job Demand-Control-Support model and previous studies, we hypothesized that the relationship between job strain (determined by job demand and control) and organizational commitment could be mediated by burnout (emotional exhaustion and depersonalization), the effect of job strain on burnout and organizational commitment could be moderated by social support. To verify the hypothesis, a quantitative cross-sectional survey was conducted, 509 OR nurses from 30 tertiary hospitals in Beijing were recruited. Multiple-group path analysis was used to test the moderated role of social support. Propensity score matching was applied to match job strain in different groups. Our research found that in the low social support group, job strain was not related to organizational commitment, while in the high social support group, depersonalization was not related to organizational commitment. Furthermore, nurses in the low social support group were more likely to have depersonalization under job strain compared to the high social support group. Social support should be provided to alleviate the negative impact of job strain.

## 1. Introduction

Hospital employees are under job strain, especially nurses [1,2]. Previous research indicated that the work environment was related to job strain [3,4]. Nurses in the operating room (OR) have unique characteristics in the work environment. OR nurses have an imbalance in job demands and job control. They have a high workload due to the growing number of surgeries, long hours of operation [5], and personnel loss [6]. Also, they have to provide continuous service and be highly concentrated throughout the procedures to ensure patient safety, in lest of the occurrence of adverse events. While, they must adapt to anesthesiologists and surgeons throughout the procedures [5], which means low job control. Furthermore, OR nurses are considered to be at high risk of occupational hazard, especially biological threat due to the exposure of patients’ blood and body fluid through sharp injuries, pricking wounds, and splashes [7,8]. In addition, the department of OR is a specialized environment where high aseptic requirements are observed, nurses in OR work in a closed windowless environment. Unique environment, coupled with high intensity, overload, low job control, and high risk might put OR nurses in a state of high job strain. 

### 1.1. Related Literature

According to previous studies, when nurses were under job strain, they were prone to turnover intention [9,10,11,12,13], less enthusiasm for work, lower job satisfaction [11,14], or poor mental health [15]. Moreover, research also showed that increased job strain led to increased burnout [9,11]. These negative influences caused by job strain will negatively affect patient safety and care quality [16]. 

Burnout is defined as a prolonged response to chronic interpersonal and emotional stressors at work [17]. It is a personal stress experience embedded in complex social relationships. According to Maslach [18], burnout is manifested by emotional exhaustion (describes the phenomenon in which individuals feel used up or drained when they are overdrawn of physical and emotional resources, especially when they are overloaded), depersonalization (refers to cynical, negative, ruthless or excessively detached reactions to others), and reduced personal accomplishment (describes the phenomenon in which individuals feel declining in productivity and incompetence, such as low morale, lack of accomplishment). Depersonalization usually occurs after emotional exhaustion, a kind of self-protective response to emotional exhaustion. Maslach and Leiter [19] suggested that emotional exhaustion and depersonalization must be contained in the measurement of burnout, and prolonged emotional exhaustion can lead to depersonalization. Previous research on nurses’ burnout indicated that workload [20], job strain [9,10,11], morally injurious events [21], anxiety, and depression [22] positively affected burnout. Nurses with high levels of burnout were more prone to have higher turnover intention [23,24], lower job satisfaction [25] and organizational commitment [26]. 

Organizational commitment is defined as the identity and involvement of an individual in the organization [27]. It implies the employee’s decision to continue to stay in the organization. According to Allen and Meyer [27], organizational commitment comprises three distinct components: affective commitment, continuous commitment, and normative commitment. Affective commitment refers to the emotional dependence, identity, and investment of employees in the organization. Continuous commitment refers to employees’ recognition of the loss caused by leaving the organization, which is a commitment that employees must stay in the organization not to lose the treatment they have earned for years of investment. Normative commitment reflects the employee’s commitment to continue to stay in the organization based on social responsibility. 

In terms of relationships among job strain, organizational commitment, and burnout, a study on nursing managers indicated that job strain could positively predict emotional exhaustion; emotional exhaustion could positively predict depersonalization. Meanwhile, depersonalization could negatively predict organizational commitment [9]. We assume that this applies to OR nurses; thus, our first hypothesis was that emotional exhaustion and depersonalization play a serial mediated effect between job strain and organizational commitment.

### 1.2. Theoretical Framework

The Job Demand-Control-Support (JDCS) model is a widely used model that focuses on relationships between work-related stresses and adverse outcomes in physiology (cardiovascular disease, sleepiness, and musculoskeletal complaints), psychology (burnout), and organization (job satisfaction, organizational commitment, and turnover intention) [28,29,30,31]. According to this model, job strain comes from the combined influence of two key aspects, job demands and job control [32]. Job demands refer to factors that reflect the number and difficulty of the tasks performed by the employees in the work environment, such as the workload, role conflict, etc. Job control demonstrates the extent to which employees can control their work behavior or the range of work decisions. Job control contains two components, skill discretion (whether the job supports innovation, develops unique skills, is possible to learn new practices and non-repetitive) and decision authority (freedom of employees to make decisions on their work and influence organizational policy and workgroup). The JDCS model has two hypotheses, the strain hypothesis and the buffer hypothesis. The strain hypothesis showed that employees with high job strain (high demand-low control) were more likely to have physical and psychological problems. According to the buffer hypothesis, social support (which refers to perceived comfort, happiness, respect, or help from others or groups) can moderate the adverse effects of high job strain on physical and mental health [33]. The literature review indicated that the strain hypothesis had considerable support from empirical research, while research on social support’s moderated role was limited and received inconsistent support due to the different composition of the participants and different outcome variables (e.g., anxiety, depression, job satisfaction, exhaustion, life satisfaction, job related-mood) [28]. However, the literature on the moderated role of social support between job strain and organizational commitment among nurses is rare. Based on the JDCS model, we proposed the second hypothesis: social support can moderate job strain on burnout and organizational commitment among OR nurses. 

### 1.3. Research Gap and Hypothesis

**Figure 1:**
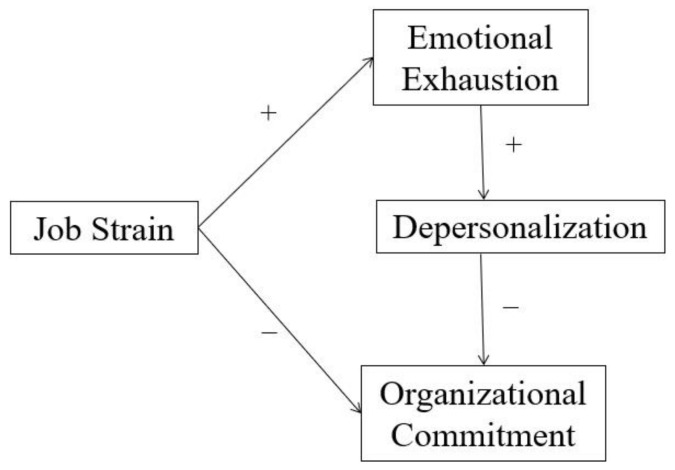
Hypothetical model.

To our knowledge, there is limited research exploring the influence path of OR nurses’ job strain on organizational commitment and burnout, and the moderated effect of social support between job strain and its adverse effects on mental health. Based on previous studies and the JDCS model, we hypothesized that the relationship between job strain and organizational commitment could be mediated by burnout (emotional exhaustion and depersonalization) (Figure 1); and the influence of job strain on organizational commitment and burnout could be moderated by social support. We aimed to testify the assumptions.

## 2. Materials and Methods

### 2.1. Research Design, Setting, and Sample

This study was a quantitative cross-sectional design that complies with the STROBE requirements. It was carried out in ORs in Beijing, China. First, hospitals were selected using a random sampling method. We got all the tertiary hospitals from the government website, then listed and numbered them. Ultimately, 30 tertiary hospitals were included using the random number table. The OR nurses from the 30 hospitals were then selected using the convenience sampling method. Registered full-time nurses who had worked for more than one year were recruited. The exclusion criteria were disagreed to participate in the survey.

### 2.2. Procedure

After the Institutional Review Board’s consent, we communicated the 30 nursing managers of OR to clarify the significance and purpose of the research, confirm the date of this investigation. Before this study, we trained one staff in each hospital. The questionnaires were given to nurses among OR in sealed envelopes. All envelopes contained a letter providing information on the purpose, significance, and anonymity of the survey, accompanied by unified instructions on the questionnaires. When the questionnaires were returned, the trained staff collected them and mailed them back to us by postal express. The survey was conducted from 18 March to 30 June 2017. Altogether, we sent 754 paper questionnaires, 569 nurses returned the questionnaires, 509 were effective for analyze, and effective return ratio was 67.5%. 

### 2.3. Study Instruments

The sociodemographic data of OR nurses consisted of age, gender, working experience in the hospital and OR, and educational level.

Job strain was assessed by *the Chinese version of the Job Content Questionnaire* [34]. It is a 3-dimension instrument that includes job demands (5 items), job control (9 items), and social support (8 items), with each item measured on a 4-point Likert scale (1 = strongly disagree, 2 = disagree, 3 = agree, 4 = strongly agree). The demand/control ratio was used to assess OR nurses’ job strain, which was calculated as follows [35]: demand/control ratio = job demand scores/job control scores × correction factor (correction factor = 12/24 = 0.5, which was the factor that corrected for the difference between the weighted number of items in the two dimensions. The demand/control ratio greater than one means high job demand-low control, which suggests the state of job strain. A higher demand/control ratio indicates a higher job strain. A higher score of social support means higher social support. The Cronbach’s alpha of the total scale and the 3 dimensions are 0.78, 0.56, 0.71 and 0.82, respectively [28]. In this study, Cronbach’s alpha of the total scale and the 3 dimensions was 0.78, 0.52, 0.67 and 0.89, respectively. 

OR nurses’ burnout (emotional exhaustion and depersonalization) was assessed with *the Chinese version of the Maslach Burnout Inventory-General Survey* [36]. The emotional exhaustion (5 items) and depersonalization (4 items) dimensions was used in this study. Items are scored on a 7-point Likert scale from 0 (never) to 6 (very often). Emotional exhaustion and depersonalization are positively scored, with a higher score indicating more significant burnout. The dimension score is the mean score of the items. The internal consistency (Cronbach’s alpha) are 0.88 and 0.83, respectively [37]. In this study, the internal consistency reached 0.96 and 0.88, respectively. 

According to Meyer et al. [38], organizational commitment can be measured by affective commitment. Thus, the *Chinese version of the Affective Commitment Scale* [39] was used to measure the participants’ organizational commitment. It was part of Meyer and Allen’s [34] *three Component organizational commitment Scale.* It is a six self-rated items instrument, with each item scored on a 5-point Likert scale from 1 (totally incompatible) to 5 (totally compatible). The score (range from 1 to 5) was the six items’ mean score, with a higher score indicating higher affective commitment. The internal consistency (Cronbach’s alpha) is 0.69 [40]. In this study, the internal consistency reached 0.84.

### 2.4. Statistical Analysis 

IBM SPSS Statistics for Windows, version 22.0 (IBM Corp., Armonk, NY, USA) was used to enter and analyze the data. Descriptive statistics were used to describe demographic characteristics, job strain, burnout, and organizational commitment. Correlation analysis was used to explore the relations between the main study variables. In all these analyses, the statistical significance level was 0.05 and two-tailed. 

The mediated effect and moderated effect were tested using IBM SPSS Amos 21.0. The bootstrap method was used to test the significance of the effects between parameters, 95% confidence interval (*CI*) excluding 0 was considered to be statistically significant. The moderated effect of social support was tested by the multiple-group path analysis [41]. Firstly, all OR nurses were classified into three groups (low, middle, and high group, *n* = 196, 152, and 161) based on the tertiles of social support. The scores of job strain were compared between the low and high social support groups with used the Student *t* test. During the comparison, the mean score of job strain in the low social support group was significantly higher than that of the high social support group (mean score of job strain = 1.40 and 1.13, respectively). Thus, a 1:1 propensity score matching was applied to match the job strain in these two groups. After using the propensity score matching, 99 pairs of nurses remained (mean score of job strain = 1.25 and 1.25, respectively). So, the 99 pairs of nurses were included in the later multiple-group path analysis. There were 4 parameters that needed to be estimated in each group, 99 participants were statistically sufficient [42].

During the multiple-group path analysis, we first tested the model among all nurses. After the model has a good fit, the model was tested in the low and high social support groups. First, constrain the structural weights, residuals, and covariances, respectively. Subsequently, calculate the difference of *χ*^2^ (_Δ_*χ*^2^) and *df* (_Δ_*df*) between the unconstrained and constrained model. If the _Δ_*χ*^2^ is significantly at the _Δ_*df* (*p* < 0.05), the moderation effect is significant. The indexes used are as follows: *χ*^2^, *p*, *df*, *χ*^2^/*df*, goodness-of-fit index (GFI), adjusted goodness-of-fit index (AGFI), comparative fit indexes (CFI), root mean square error of approximation (RMSEA) [43,44]. 

## 3. Results

### 3.1. Descriptive Statistics

The mean age of OR nurses was 35.41 ± 6.87 years, ranging from 22 to 55 years. Among them, 92.9%were female. The main educational level was bachelor degree (76.0%). The mean years of working experience in the hospital was 14.70 ± 7.70 years, and the mean years of working experience in the OR was 14.36 ± 7.70 years, both range from 1 to 34. The descriptive statistics for the main study variables are shown in Table 1. The mean score of job demand, job control, and social support was 37.26 ± 4.65 (range 24–48), 60.64 ± 8.63 (range 30–94), and 23.96 ± 4.13 (range 13–32), respectively. The score of demand/control ratio was 1.26 ± 0.27 (range 0.57–2.71), and 409 OR nurses (80.4%) had a ratio greater than 1. 

### 3.2. Correlation between Study Variables 

The correlation analysis between job strain, organizational commitment, emotional exhaustion, and depersonalization are shown in Table 1. Emotional exhaustion was positively related to depersonalization (*r* = 0.702, *p* < 0.001), with a strong effect. Other variables were correlated with a moderate effect. 

### 3.3. Model Test

**Figure 2:**
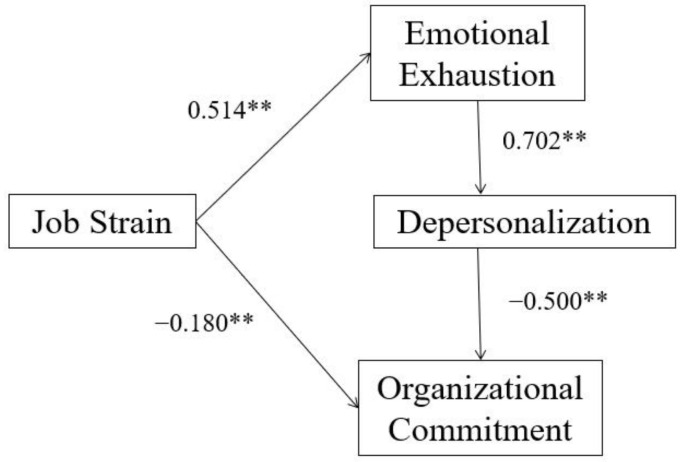
Final model (*n* = 509, R^2^ = 0.348). ** *p* < 0.01 (2-tails).

**Figure 3:**
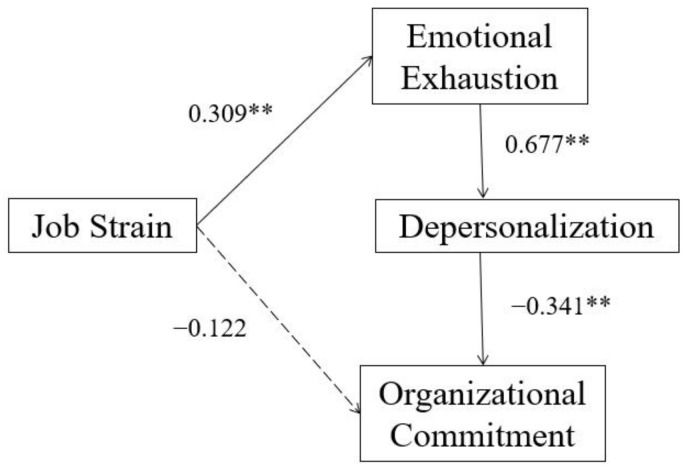
Low social support group (*n* = 99, R^2^ = 0.234). ** *p* < 0.01 (2-tails); dotted line means unsignificant coefficients.

**Figure 4:**
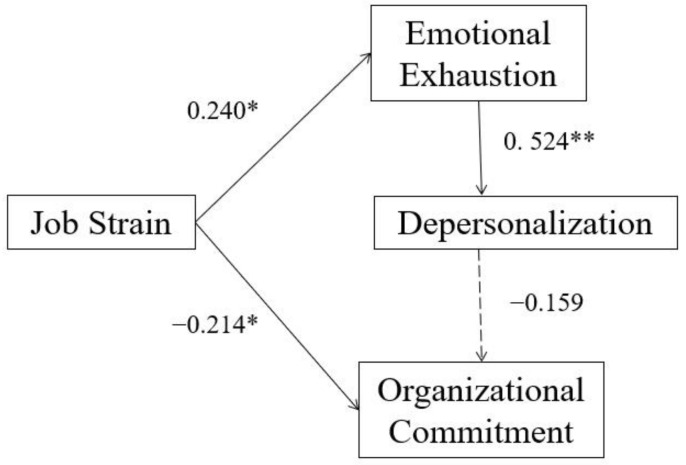
High social support group (*n* = 99, R^2^ = 0.193). * *p* < 0.05; ** *p* < 0.01 (2-tails); dotted line means unsignificant coefficients.

The results of our hypothetical model to test the relationships among job strain, organizational commitment, and burnout, and the moderated role of social support are shown in Figure 2, Figure 3 and Figure 4, and Table 2 and Table 3. The effects are presented in Table 4. Table 2 demonstrated that all the participants (*n* = 509), low (*n* = 99), and high (*n* = 99) groups of social support had a good model fit (*p* > 0.05). Table 3 demonstrated that the _Δ_χ^2^ was significantly different at the _Δ_*df* (*p* < 0.05) between the high and low social support groups, which supported our hypothesis that social support could moderate the effect of job strain on burnout and organizational commitment. To be more specific, among path coefficients with significance, only the path coefficient between emotional exhaustion and depersonalization has significant difference between two groups according to the critical ratios (CR) for differences between parameters of multiple-group analysis (CR = |−3.757| > 1.96). Table 4 and Figure 2 demonstrated that job strain positively predicted emotional exhaustion (*β* = 0.514). Emotional exhaustion positively predicted depersonalization (*β* = 0.702) in all nurses. Depersonalization negatively predicted organizational commitment (*β* = −0.500). Moreover, job strain had a direct effect (*β* = −0.180) on organizational commitment and an indirect negative effect (*β* = −0.181) on organizational commitment via emotional exhaustion and depersonalization, the total effect was −0.361, and it explained 34.8% of the variance in organizational commitment.

## 4. Discussion

As assumed, the results supported our hypothesis on the moderated role of social support among job strain, organizational commitment, and burnout. The findings expanded the limited understanding of the job strain of nurses in OR in China and its influence on burnout and organizational commitment.

The result showed that 80.40% of participants had a demand/control ratio greater than 1, indicating that the job strain of nurses in OR was higher than general nurses in China [35,45]. Trousselard [46] showed that the level of job strain among OR nurses was at the ‘high pressure’ group. A study in China showed that nurses in OR considered the leading cause of high job strain arisen from workload and time pressure [5]. Another survey of OR nurses in Finland also showed that workload was the leading cause of job strain [47]. This might due to the unique characteristics of nursing practice in OR. OR nurses, especially the instrument nurses, must carry on continuous work during operations. They cannot predict when the operation is over, and there might be accidents at any time during the operation. The emergence of new surgical techniques also brought new challenges to OR nurses [48]. So, nurses in OR have a higher job demand. According to Karasek’ [32] JDCS model, nurses with high job demand and low job control were under job strain. The OR nurses had perception of low autonomy or control than general nurses. During operations, they have to adapt to different techniques and personality characteristics of surgeons and anesthesiologists [5]. Thus, our results showed that nurses in OR had more job pressure. 

According to our finding, job strain was directly negatively related to emotional exhaustion; emotional exhaustion was directly positively related to depersonalization; depersonalization was directly negatively related to organizational commitment. Moreover, job strain was directly negatively associated with organizational commitment. In other words, emotional exhaustion and depersonalization played a serial mediated effect between job strain and organizational commitment (*β* = −0.181). Although the relationship between job strain and burnout was consistent with previous studies [9,10,11], the serial mediated role of emotional exhaustion and depersonalization between job strain and organizational commitment among OR nurses was unique to our study. Due to the particularity of OR service we mentioned in ‘Introduction’, nurses in OR are under greater pressure than general nurses both physically and psychologically [49]. Long-term job strain can consume nurses’ energy gradually; resulting in feelings of excessive exhaustion or lack of energy and enthusiasm for job, energy loss, which in turn leads to depersonalization [50] (such as indifferent, negative attitude to co-workers or patients) and declining organizational commitment (such as decreased emotional dependence or recognition of organizational values). 

The multiple-group path analysis showed that social support could moderate the effect of job strain on organizational commitment and burnout, which was consistent with the second hypothesis we proposed according to the JDCS model. Propensity score matching was applied to match job strain in the low and high social support groups. So, the difference in the path coefficients between the two groups was resulted from differences in social support rather than differences in job strain. In the low social support group, job strain was not related to organizational commitment, while in the high social support group, depersonalization was not related to organizational commitment. 

Our findings demonstrated that the path coefficient from emotional exhaustion to depersonalization was significantly different (*β* = 0.677 vs. 0.524) between the low and high social support groups, which means that when OR nurses were under job strain, those who have low social support were more likely to have depersonalization. Social support includes support from co-workers and supervisors. Literature review in China indicated that the policies of the hospital, the organizational atmosphere and the support of the co-workers or supervisors could effectively alleviate occupational strain and burnout of nurses [51]. A longitudinal study demonstrated that social support from colleagues and supervisors improved nurses’ work meaning, which in turn alleviated burnout [23]. 

Based on this finding, OR nurses should be given more social support to alleviate the negative impact of job strain on depersonalization. OR nurses mainly worked in OR and completed surgery with anesthesiologists and surgeons. Thus, the support from their peers on knowledge and skills, establishing a good interpersonal relationship with their partners through a long period of cooperation, and the support from supervisors with good leadership are helpful [23,41,51]. 

The survey had several limitations. Since the survey was cross-sectional, causal relationships were impossible to be inferred. Moreover, the results of our study might not be extrapolated to all OR nurses in China due to the convenient sampling conducted in Beijing. Third, selection bias or reporting bias might be generated due to the low effective response rate and the self-reported data collection method. Last, using the Affective Commitment Scale to measure organizational commitment might cause measurement bias.

## 5. Conclusions

OR nurses in China were under high job strain. Based on the JDCS model, the results provided empirical support for our hypothesis. This study demonstrated that when nurses were under job strain, they could have emotional exhaustion, which in turn leads to depersonalization, and finally causes lower organizational commitment. On the other hand, job strain can lead to low organizational commitment directly. Moreover, emotional exhaustion and depersonalization played a serial mediated role between job strain and organizational commitment. Social support moderated the relationships among job strain, emotional exhaustion, depersonalization, and organizational commitment.

### 5.1. Theoretical Implications

This study clarified the serial mediated role of emotional exhaustion and depersonalization in the relationship between job strain and organizational commitment, and the moderated role of social support in the relationship between job strain, burnout, and organizational commitment, which provide empirical support for related theories and reference for future research.

### 5.2. Practical Implications

Based on our findings, nursing managers should pay more attention to the job strain (imbalance between job demand and job control) of OR nurses. Reducing operating room nurses’ job strain might be helpful in easing emotional exhaustion and depersonalization, and improving organizational commitment. Moreover, social support from co-workers and supervisors should be provided to alleviate the negative impact of job strain.

### 5.3. Further Research

The random cluster sampling method and the nationwide survey are necessary for further research. Longitudinal studies should be conducted to confirm the serial mediated role of emotional exhaustion and depersonalization, as well as the moderated role of social support among nurses and other occupations. Specific interventional studies should be carried out.

## Figures and Tables

**Table 1:** Descriptions and correlations of the main variables (*n* = 509).

Variable	*Mean*	*Median*	*SD*	*Range*	1	2	3	4
Job demand	37.26		4.65	24–48				
Job control	60.64		8.63	30–94				
Social support	23.96		4.13	13–32				
1. Job strain (Demand/control ratio)	1.26		0.27	0.57–2.71	1.000			
2. Emotional exhaustion	3.32	3.40	1.83	0–6	0.514 ***	1.000		
3. Depersonalization	1.98	1.25	1.88	0–6	0.361 ***	0.702 ***	1.000	
4. Organizational commitment	3.67		0.86	1–5	−0.360 ***	−0.444 ***	−0.565 ***	1.000

Note: *** *p* < 0.001 (2-tails).

**Table 2:** The results of simultaneous analysis of several groups.

Model	*χ* ^2^	*p*	*df*	χ^2^/*df*	GFI	AGFI	CFI	RMSEA
M_all_ (*n* = 509)	5.346	0.069	2	2.678	0.992	0.974	0.995	0.057
M_high_ (*n* = 99)	1.574	0.455	2	0.787	1.000	0.960	1.000	<0.001
M_low_ (*n* = 99)	2.496	0.287	2	1.248	0.988	0.939	0.994	0.05
M1	4.070	0.397	4	1.017	0.990	0.949	0.999	0.009
M2	18.489	0.018	8	2.311	0.958	0.894	0.915	0.082
M3	18.490	0.030	9	2.054	0.958	0.906	0.923	0.073
M4	25.830	0.011	12	2.152	0.943	0.904	0.888	0.077

M1 = Unconstrained; M2 = Structural weights; M3 = Structural covariances; M4 = Structural residuals; Note: GFI = goodness-of-fit index; AGFI = adjusted goodness-of-fit index; CFI = comparative fit indexes; RMSEA = root mean square error of approximation.

**Table 3:** Comparison of nested model.

Model	*df*	*χ* ^2^	*p*	NFI Delta-1	IFI Delta-2	RFI rho-1	TLI rho-2
Structural weights constrained	4	14.419	0.006	0.107	0.110	0.115	0.126
Structural covariances constrained	5	14.420	0.013	0.107	0.110	0.092	0.101
Structural residuals constrained	8	21.760	0.005	0.161	0.166	0.101	0.110

Note: NFI = normed fit index; IFI = incremental fit index; RFI = relative fit index; TLI = Tacker-Lewis index.

**Table 4:** Model Effects.

		Directed Effects		Indirected Effects		All Effects	
		*β*	95% *CI*	*β*	95% *CI*	*β*	95% *CI*
Job strain →emotional exhaustion	All	0.514	0.453~0.571 **	-	-	0.514	0.453~0.571 **
	High group	0.240	0.013~0.456 *	-	-	0.240	0.013~0.456 *
	Low group	0.309	0.138~0.470 **	-	-	0.309	0.138~0.470 **
Job strain →depersonalization	All	-	-	0.361	0.308~0.411 **	0.361	0.308~0.411 **
	High group	-	-	0.126	0.005~0.265 *	0.126	0.005~0.265 *
	Low group	-	-	0.209	0.090~0.320 **	0.209	0.090~0.320 **
Job strain →organizational commitment	All	−0.180	−0.264~−0.084 **	−0.181	−0.223~−0.143 **	−0.361	−0.434~−0.273 **
	High group	−0.214	−0.404~−0.021 *	−0.020	−0.061~0.003	−0.234	−0.414~−0.052 *
	Low group	−0.122	−0.297~0.046	−0.171	−0.136~−0.024 **	−0.194	−0.360~0.024 *
Emotional exhaustion →depersonalization	All	0.702	0.659~0.741 **	-	-	0.702	0.659~0.741 **
	High group	0.524	0.345~0.656 **	-	-	0.524	0.345~0.656 **
	Low group	0.677	0.564~0.772 **	-	-	0.677	0.564~0.772 **
Emotional exhaustion →organizational commitment	All	-	-	−0.351	−0.412~−0.290 **	−0.351	−0.412~−0.290 **
	High group	-	-	−0.083	−0.190~0.013	−0.083	−0.190~0.013
	Low group	-	-	−0.231	−0.355~−0.105 **	−0.231	−0.355~−0.105 **
Depersonalization →organizational commitment	All	−0.500	−0.576~−0.420 **	-	-	−0.500	−0.576~−0.420 **
	High group	−0.159	−0.353~0.023	-	-	−0.159	−0.353~0.023
	Low group	−0.341	−0.503~−0.166 **	-	-	−0.341	−0.503~−0.166 **

Note: * *p* < 0.05 (2-tails); ** *p* < 0.01 (2-tails).

## Data Availability

The data presented in this study are available on request from the corresponding author.
